# Transient middle cerebral artery occlusion induces microglial priming in the lumbar spinal cord: a novel model of neuroinflammation

**DOI:** 10.1186/1742-2094-5-29

**Published:** 2008-07-07

**Authors:** Katie Moisse, Ian Welch, Tracy Hill, Kathryn Volkening, Michael J Strong

**Affiliations:** 1 Cell Biology Research Group, Robarts Research Institute, London, Ontario, Canada; 2 Department of Pathology, University of Western Ontario, London, Ontario, Canada; 3 Department of Animal Care and Veterinary Services, University of Western Ontario, London, Ontario, Canada; 4 Department of Clinical Neurological Sciences, the University of Western Ontario, London, Ontario, Canada

## Abstract

**Background:**

Middle cerebral artery occlusion (MCAo) in mice results in a brain infarct, the volume of which depends on the length of occlusion. Following permanent occlusion, neuropathological changes – including a robust glial inflammatory response – also occur downstream of the infarct in the spinal cord.

**Methods:**

We have performed short, transient MCAo in mice to induce penumbral damage spanning the motor cortex. A 30 minute MCAo using a poly-L-lysine-coated intraluminal suture introduced through a common carotid artery incision was performed in 17 female C57BL/6 mice. Five sham-operated mice received common carotid artery ligation without insertion of the suture. Neurobehavioural assessments were performed during occlusion, immediately following reperfusion, and at 24 and 72 hours post-reperfusion. Routine histological and immunohistochemical studies were performed at 24 and 72 hours.

**Results:**

In 11 of the surviving 16 mice subjected to MCAo, we observed a focal, subcortical necrotic lesion and a reproducible, diffuse cortical lesion with accompanying upper motor neuron involvement. This was associated with contralateral ventral spinal cord microglial priming without significant reactive astrocytosis or lower motor neuron degeneration.

**Conclusion:**

The advantages to this method are that it yields a reproducible cortical lesion, the extent of which is predictable using behavioural testing during the period of ischemia, with upper motor neuron involvement and downstream priming, but not full activation, of microglia in the lumbar spinal cord. In addition, survival is excellent following the 30 minutes of occlusion, rendering this a novel and useful model for examining the effects of microglial priming in the spinal motor neuron pool.

## Background

Neuroinflammation is a pathological feature of several neurodegenerative conditions including amyotrophic lateral sclerosis (ALS), which involves the selective degeneration of motor neurons in the brain and spinal cord. There is controversy over the role of neuroinflammation in the disease process. Microglia become activated and proliferate in areas of neurodegeneration with disease progression in human patients and in animal models of the disease [1,2]. Anti-inflammatory therapies have shown efficacy in mouse models of ALS [3-6], and activated microglia can have cytotoxic effects on motor neurons in culture [7-10]. Microglial activation is a response to damage signals from neurons and astrocytes. This activation is seen morphologically as a transition from a resting, ramified state to an active, amoeboid state through a "*primed*" intermediate state [11].

Once activated, microglia have cytotoxic and phagocytic potential. However, the role of primed microglia in the early response to damage signals remains unclear. Primed microglia express major histocompatibility class (MHC) II molecules and have antigen presenting capabilities. Priming results in intensification of local surveillance and production of pro- or anti-inflammatory cytokines [12]. It is possible that microglia perform different tasks depending on whether they are "primed" or "activated". We predict that the function of *primed *microglia is to protect the damaged neuron from further injury and enable recovery, while *activated *microglia serve to remove the damaged neuron in order to preserve proper function of surrounding cells. In order to examine this, we have developed a model in which the function of primed microglia can be studied. Ultimately, we intend to use this model to examine the effects of microglial priming on healthy motor neurons and on motor neurons predisposed to develop ALS-like pathology.

The induction of cerebral ischemia in animals is a commonly used method to investigate the pathophysiology of stroke. A relatively non-invasive procedure has been developed in rats and involves the insertion of an intraluminal suture into the Circle of Willis to occlude the middle cerebral artery (MCA) [13]. The suture can be removed after a period of occlusion resulting in reperfusion of the Circle of Willis and the production of a marked region of infarct. This method has been modified for use in mice [14,15]. However, this latter model suffers from a high rate of mortality and inconsistencies in stroke outcome, including high variability of lesion size. Because variability in animal size, strain, and cerebrovascular anatomy can directly affect the consistency of stroke outcome in mice, modifications that include coating the suture material with poly-L-lysine to enhance adhesion of the suture to the vascular endothelium [16] and increasing occlusion time to maximize infarct volume [17,18] have been introduced. When coupled with a neurobehavioural assessment, an accurate prediction of lesion severity can be made during MCAo [17], thus allowing for the exclusion of animals that are unlikely to harbour the necessary infarct from further study. MCAo of 60 minutes or less leads to a substantial recovery of function within 24 hours [17]. While longer occlusion times result in persistence of behavioural symptoms including altered reflexes and contralateral weakness, they are also associated with increased mortality [15,19].

Most of the studies examining the cellular effects of cerebral ischemia have focused on the primary lesion in the brain. However, neuropathological changes occur far removed from the focal lesion epicentre in studies carried out in rats [20,21]. These changes include a glial inflammatory response in the contralateral lumbar spinal cord 24 hours following permanent MCAo in which ventral horn motor neurons that appear to be undergoing degeneration are engulfed by phagocytic microglia [20]. This is accompanied by an increase in expression of pro-inflammatory cytokines and markers of oxidative stress 24–72 hours following permanent MCAo [21,22]. The mechanism by which this inflammatory response is induced is unknown. However, it has been suggested that it may be due to transsynaptic degeneration mediated by ischemic degeneration of the descending supraspinal (upper motor neuron) pathways and an associated deafferentation of the ventral spinal motor neurons [20]. This in turn is postulated to give rise to a glutamatergic injury of the postsynaptic lower motor neuron through release of presynaptic glutamate, the activation of postsynaptic N-methyl-D-aspartate (NMDA) receptors, and an accompanying increased calcium influx [23]. The MCAo model has therefore provided a unique opportunity to observe the effects of upper motor neuron injury on lower motor neurons and surrounding glia in the rat.

In these experiments, we have developed a model of murine MCAo that is easily quantifiable, reproducible in terms of upper motor neuron involvement regardless of the overall size of the cerebral infarct, and which gives rise to microglial priming in the contralateral lumbar ventral horn 24 hours and 72 hours post-reperfusion in mice. We have observed that a 30 minute occlusion time was associated with 100% survival at the 24 hour post-reperfusion time interval. We propose that this model, easily performed in mice, will allow for the study of microglial priming in the lumbar spinal cord in a reproducible manner that will be instrumental in determining the exact roles of early microglial priming and activation, an event that can apparently produce two very opposing outcomes following neuronal injury.

## Methods

### Animals

Female C57BL/6 mice were originally purchased from Charles River Laboratories (Montreal) and bred in house. Studies were carried out on mice aged six weeks, weighing 17–22 g independently of the estrous cycle. Cyclic changes in estrogen levels may explain the relatively large variability in the number of apoptotic neurons detected in the brain following MCAo. Mice were kept in group cages and allowed free access to food and water throughout the duration of the study. All procedures were in accordance with the Canadian Council for Animal Care and the University Council on Animal Care guidelines for research, and all protocols involving animals, behavioural testing, surgeries and animal maintenance were approved in accordance with the above guidelines.

### Surgical preparation and middle cerebral artery occlusion

The tip of the 5-0 monofilament suture to be used in the MCAo was first blunted by heating over a flame and then the distal 11 mm coated with poly-L-lysine (Sigma) (0.1% in deionized water) and dried in a 60°C oven for 1 hour. A point 11 mm from the blunt end was marked with silver impregnation marker to render it visible through the arterial wall.

Mice were placed in an induction chamber supplied with oxygen at a rate of 1 L/min and isofluorane at 4%. Injectable chemical anaesthesia was avoided in order to accelerate post-surgical awakening for immediate behavioural observation. All mice were maintained on 1 L/min oxygen supplemented with 1.5% isofluorane throughout the duration of surgery using a small mask. Using an operating microscope, the left common carotid artery (CCA) was exposed through a midline neck incision and dissected free from surrounding tissue from 1 cm proximal to its bifurcation to the base of the skull. The external carotid artery (ECA) was dissected and coagulated near the origin of the lingual and maxillary artery branches. The internal carotid artery (ICA) was isolated and separated from the adjacent vagus nerve. A 5-0 vicryl suture was tied around the CCA 1 cm proximal to the bifurcation. The 2 cm poly-L-lysine-coated suture was then inserted via a small incision in the CCA distal to where it was tied, but proximal to the bifurcation, and advanced 11 mm from the CCA bifurcation to occlude the middle cerebral artery (MCA). A 5-0 vicryl suture was placed around the ICA and intraluminal nylon suture to prevent bleeding and movement of the nylon suture. The neck skin was closed with 5-0 vicryl sutures.

The mice were placed in separate cages and allowed to awaken briefly from anaesthesia during the 30 minutes of occlusion to permit neurobehavioural assessment. Following assessment, the mice were re-anaesthetized and after 30 minutes of occlusion, the neck skin was re-opened, the nylon occlusion suture carefully removed, and the neck skin closed with 5-0 vicryl sutures.

In this method, and in contrast to the procedure reported by Belayev and colleagues [17], the occipital artery branches of the external carotid artery and the pterygopalentine artery were not coagulated or ligated. Kitagawa and colleagues [15] showed that differences in cranial vasculature, specifically the posterior communicating artery, could affect the degree of ischemia and should be assessed during occlusion by measuring cortical microperfusion. Because we correlated neurobehavioural testing during the occlusion with the ischemic lesion volume, this more technically challenging evaluation could be avoided.

The mice were allowed to recover in separate cages and given antibiotics (0.01 ml ampicillin, 50 mg/kg) and analgesics (0.04 – 0.05 ml buprenorphine, 0.09 mg/kg) post-surgery. Animals were also given up to 4 mL daily subcutaneous fluids throughout the recovery period and provided with a high peptide liquid nutritional supplement (Peptamen) and rice pabulum on the cage floor for the duration of post-operative study. The criterion for early euthanasia was adhered to in the event of excessive weight loss.

Sham-operated mice were prepared for surgery as above. The left CCA was exposed through a midline neck incision, dissected free from surrounding tissue, and ligated with a 5-0 vicryl suture 1 cm proximal to the bifurcation. The neck skin was closed with 5-0 vicryl sutures.

### Neurobehavioural assessment

Neurobehavioural evaluations consisted of two testing batteries and were performed during occlusion (20 minutes after suture insertion), following recovery from reperfusion, 24 hours, and 72 hours post-reperfusion. The first test battery consisted of 2 test items each scored on a scale from 0–12 (0 = normal, 12 = severely impaired) [17]: the postural reflex test to observe the animal's upper body posture while being suspended by the tail [24]; and the forelimb placing test to examine psychomotor responses to tactile stimuli [25]. For the second battery, we attempted to assess the motor function of each mouse without handling. Each mouse was assigned a score of 0 – 4: 0 = no observable neurological deficit; 1 = failure to extend the right forepaw; 2 = circling to the right; 3 = falling to the right; and 4 = cannot walk spontaneously [26].

### Tissue preparation and lesion assessment

Based on the rat MCAo studies [21], we examined the spinal cord 24 hours after reperfusion for the extent microglial proliferation or activation and effects on lower motor neurons. Mice were anaesthetized 24 hours (n = 8) or 72 hours (n = 8) after reperfusion of the MCA or 24 hours following sham operation (n = 5) via intraperitoneal injection of ketamine-xylazine mixture (0.03 ml/10 g) and perfused transcardially with heparinized saline until the outflow ran clear. Fixation was achieved with 25 ml/min intracardial injection of 50 ml/100 g body weight of fixative (4% paraformaldehyde in PBS). Brains and spinal cords were dissected out and placed in the same fixative overnight at 4°C. The frontal brain and lumbar spinal cord from L3-S1 was processed for paraffin-sectioning. Five μm thick serial sections were cut in the coronal plane and mounted on glass slides. Every 5^th ^section was stained with haematoxylin and eosin (H&E), or immunoreacted using antibodies to detect macrophages (ionized calcium-binding adaptor molecule-1 (IBA-1), Wako), astrocytes (glial fibrillary acid protein (GFAP), BD Pharmingen) or apoptosis (active caspase 3, BD Pharmingen).

Sections were dried overnight, deparaffinized, and rehydrated. Antigen retrieval was performed using boiling high-pH TRIS-EDTA buffer following which the sections were blocked for endogenous hydrogen peroxide (3% hydrogen peroxide, 5 min.). Non-specific antibody binding was blocked (10% normal goat serum, 5% bovine serum albumin (BSA) in PBS containing 0.3% Triton X-100; 30 min.) and the sections then incubated with the primary antibody (1 hr, room temperature). Primary antibodies consisted of either polyclonal rabbit-anti-IBA-1 (1:1000), monoclonal mouse-anti-GFAP (1:75) or polyclonal rabbit-anti-active caspase 3 (1:500). All primary antibodies were prepared in 5% BSA, 5% normal goat serum, and 0.3% Triton X-100. Following this, sections were incubated in secondary antibody (1:200 goat-anti-rabbit or horse-anti-mouse in 5% normal goat serum, 5% BSA, 0.3% Triton X-100 in PBS) for 30 minutes at room temperature. The antigen:antibody complex was localized using the avidin-biotin complex (ABC staining kit, Vector Laboratories) as per the manufacturer's instructions. Sections were washed and developed for visualization in 0.3 mg/ml 3', 3-diaminobenzidine tetrahydrochloride (DAB, Sigma) containing 0.01% hydrogen peroxide for 10 minutes and then washed thoroughly. Sections were counterstained with Harris's Haematoxylin and then mounted in Cytoseal 60 (VWR). Negative control slides consisted of the omission of the primary antibody.

All cell counts were carried out in defined areas maintained across all sections. For the quantification of the percent IBA-1-positive area (to support the observation of an increase in the number of primed microglia), images of IBA-1-immunolabelled sections were captured using Northern Eclipse imaging software. A threshold was applied to the image which allowed for calculation of the total area of immunoreaction and the percent immunoreaction in a defined area which was consistent across all sections.

### Statistical analysis

One-way ANOVAs were performed with Tukey post-hoc tests for neuronal density, neuronal caspase 3 activation, neurobehavioural assessment analysis, IBA-1 immunoreactivity assessment, and primed microglial cell counts,.

## Results

### Induction of cerebral ischemia with upper motor neuron involvement

**Figure 1:**
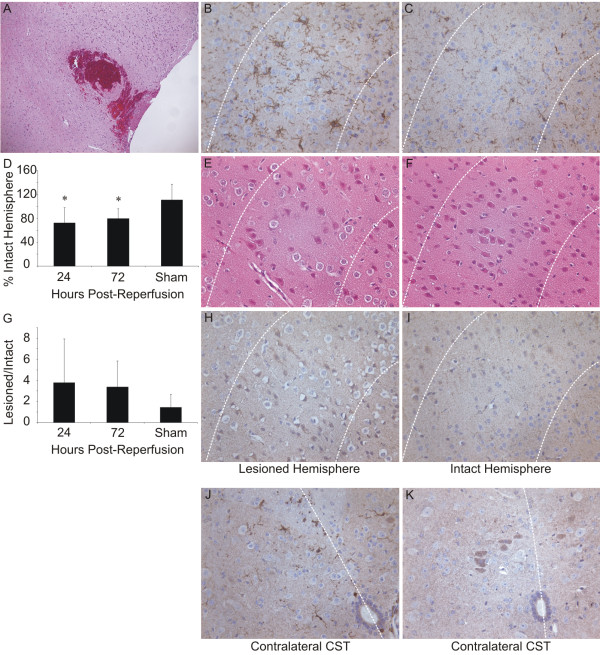
(a) The focal necrotic infarct region resulting from 30-minute transient MCAo (magnification 20×). In all cases in which a focal necrotic infarct region could be identified, it was subcortical with diffuse penumbral effects spanning cortically. (b) Evidence for penumbral damage spanning into the cortex as indicated by IBA-1 immunoreactivity (a marker for macrophages) in layer V of primary motor cortex in the lesioned hemisphere, (c) compared to the intact hemisphere. (d) Histogram depicting loss of large neurons in layer V of the motor cortex expressed as percent intact hemisphere (ANOVA p < 0.01) supported by representative images of H&E-stained sections from (e) the lesioned hemisphere and (f) the intact hemisphere. (g) Histogram depicting a trend towards caspase 3 activation in large neurons in layer V of the motor cortex expressed as fold change lesioned/intact (ANOVA, p > 0.05) supported by representative images of sections labelled with antibodies directed against active caspase 3 (BD Pharmingen) from (h) the lesioned hemisphere and (i) the intact hemisphere. Dotted white lines indicate layer V. (j) Microglial activation in the corticospinal tract contralateral to MCAo. (k) Active caspase 3-poitive neurons in the corticospinal tract contralateral to MCAo. Dotted lines in b-c, e-f, and h-i indicate layer V. Dotted lines in j-k indicate the spinal cord midline. All images taken at magnification 40× prior to reproduction unless noted.

Thirty minutes of MCAo resulted in diffuse penumbral damage spanning both the subcortex and the cortex in 5 of 8 mice at the 24-hour time point and 6 of 8 mice at the 72 hour time point. In these mice a necrotic focal lesion was clearly visible (Table 1, Figure 1a). Two of 5 mice that did not exhibit cortical pathology did demonstrate behavioural deficits during occlusion meeting the inclusion criteria proposed by Belayev and colleagues [17]. All 5 mice were excluded from further study.

**Table 1:** Summary of cases, indicating presence or absence of a focal infarct region and/or upper motor neuron (UMN) involvement, and neurobehavioural assessment (NBA) scores using the testing criteria of Belayev at al. [17] or Yang et al. [26].

**Case**	**Focal Infarct**	**UMN Involvement**	**Belayev et al. **([17]) **NBA Score**	**Yang et al. **([26]) **NBA Score**
**1-1**	+	+	12	3
**1–2**	+	+	22	4
**1–3**	-	-	1	1
**1–4**	+	+	17	2.5
**1–5**	-	-	22	4
**1–6**	-	-	8	1
**1–7**	+	+	22	2
**1–8**	+	+	12	1
**3-1**	-	-	0	0
**3-2**	+	+	24	4
**3-3**	+	+	18	3
**3–4**	-	-	1	1
**3–5**	+	+	16	3
**3–6**	+	+	20	4
**3–7**	+	+	14	2
**3–8**	+	+	16	2

In the remaining mice, all harbouring marked infarct regions, microglial activation was prominent, extending into the motor cortex of the lesioned hemisphere and not the intact hemisphere (Figures 1b and 1c). The number of large neurons in layer V of the primary motor cortex in these mice was decreased in the lesioned hemisphere compared to the intact hemisphere (Figures 1d–f, ANOVA p < 0.01). This was accompanied by an apparent increase in active caspase 3 immunoreactivity in the same region that did not reach significance due to variability (Figures 1g–i, ANOVA p = 0.1133). We observed microglial activation (Figure 1j) and caspase 3 activation (Figure 1k) in the corticospinal tract (CST) contralateral to MCAo, further suggesting the presence of an injury upstream to ventral horn motor neurons. Mice who did not display evidence of any focal infarct region did not have any pathology (glial or neuronal) in the motor cortex in either hemisphere (Table 1).

### Neurobehavioural deficits and mortality

**Figure 2:**
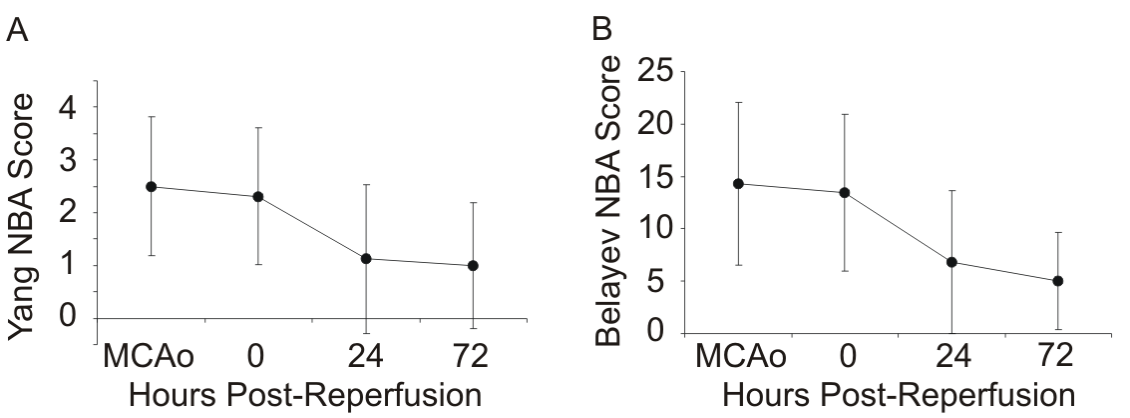
Neurobehavioural assessment (NBA) scores of mice using (a) the Belayev et al. [17] scale and (b) the Yang et al. scale [26], during occlusion (MCAo), following reperfusion (0), 24 hours post-reperfusion and 72 hours post-reperfusion. All sham-operated mice exhibited scores of 0 at all time points.

During occlusion, all mice exhibited head rotation, eyelid drooping and abnormal tongue holding consistent with the development of a cerebral infarct. Interestingly, mice were highly vocal throughout the course of the study. Animals were examined carefully for evidence of pain and were not in apparent distress. Analgesics were administered according to guidelines. Following reperfusion, behavioural deficits were sustained with 12 of 16 mice achieving scores above the inclusion cutoffs proposed by Belayev and colleagues [17] suggesting the presence of a lesion (Figure 2b). Eleven of these 12 mice had a marked region of infarct and cortical pathology. Twenty-four hours post-reperfusion most animals remained vocal with only a moderate head tilt and a mild postural reflex (Figure 2a–b). All animals survived 24 hours post-reperfusion and maintained good body weight. Eight of 9 animals survived 72 hours post reperfusion.

### Microglial priming in the contralateral lumbar ventral horn

**Figure 3:**
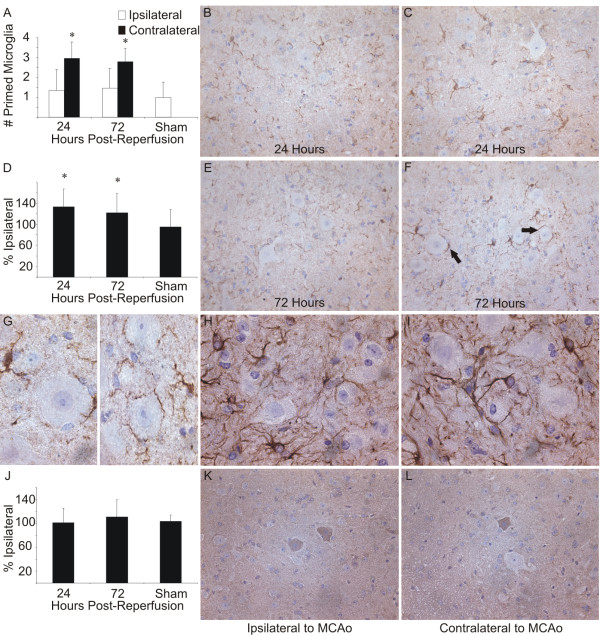
(a) Quantification of the number of IBA-1-immunolabeled microglia with primed morphology in ventral horns ipsilateral to MCAo compared to ventral horns contralateral to MCAo in mice 24 hours and 72 hours post-reperfusion and in sham-operated mice. (b) Representative image from a ventral horn ipsilateral and (c) contralateral to MCAo 24 hours post-reperfusion immunolabelled with antibodies directed against IBA-1 for visualization of microglia. (d) Quantification of the IBA-1 immunoreactivity expressed as percent ipsilateral IBA-1-immunoreacted area 24 hours and 72 hours post-reperfusion and in sham-operated mice. (e) Representative image from a ventral horn ipsilateral and (f) contralateral to MCAo 72 hours post-reperfusion immunolabelled with antibodies directed against IBA-1 for visualization of microglia. (g) Higher magnification image of microglia in close proximity to large motor neurons in ventral horns contralateral to MCAo (magnification 100×). (h) Representative image from a ventral horn ipsilateral and (i) contralateral to MCAo immunolabelled with antibodies directed against GFAP for visualization of astrocytes (magnification 100×, same section as in g). (j) Quantification of caspase 3 activation expressed as percent ipsilateral number of caspase 3-positive motor neurons 24 hours and 72 hours post-reperfusion and in sham-operated mice. (k) Representative image from a ventral horn ipsilateral and (l) contralateral to MCAo 72 hours post-reperfusion immunolabelled with antibodies directed against active caspase 3 for visualization of apoptotic cells. All images taken at magnification 40× prior to reproduction unless noted.

There were significantly more microglia with primed morphology in the ventral horn contralateral to MCAo than in the ventral horn ipsilateral to MCAo 24 hours and 72 hours post-reperfusion (Figure 3a–c, e–f, ANOVA p < 0.05). The total area of IBA-1 immunoreaction was also greater in the ventral horn contralateral to MCAo at both time points (Figure 3d, ANOVA p < 0.01). Cell bodies appeared thicker and processes were shorter (Figure 3c and 3f). Often primed and ramified microglial processes were enveloping large motor neurons (arrows in Figure 3f, higher magnification in 3g).

Despite the observed change in microglial activation state from ramified to primed, there was no difference observed in the morphology of GFAP-immunolabelled astrocytes in the ventral horns contralateral to MCAo compared with ventral horns ipsilateral to MCAo 24 hours and 72 hours post-reperfusion (Figure 3h–i). There was no significant difference observed in the number of apoptotic motor neurons in the ventral horns contralateral to MCAo compared with ventral horns ipsilateral to MCAo 24 hours and 72 hours post-reperfusion (Figure 3j–l).

## Discussion

We have observed microglial priming in the lumbar spinal cord following a contralateral MCAo of 30 minutes duration in mice. The motor cortex was included in the span of the diffuse lesion created by temporary MCAo. Loss of larger neurons in layer V of the motor cortex was confirmed and neuronal apoptosis in the same region was observed. Despite this inflammatory response, we did not observe morphological evidence of reactive astrocytosis or caspase 3 activation in spinal motor neurons 24 hours and 72 hours post-reperfusion. Based on morphological criteria, microglia were primed and not fully activated. However, the functional differences between primed microglia in this model and microglia in models of activation remain to be determined.

Nerve lesion models have been useful in the study of the effects of inflammation, executed primarily by microglia, on injured neurons. Microglial activation can induce neuronal repair through the release of anti-inflammatory cytokines, neurotrophins and growth factors, and through controlled phagocytosis of neurons damaged beyond repair. Conversely, the response can be to enhance neuronal injury through the release of pro-inflammatory cytokines, reactive oxygen and nitrating species, and lysosomal proteases, and through chronic phagocytosis [12]. Here we report that a model of transsynaptic injury, in which there is no direct injury to lower motor neurons, results in a reproducible response of microglial priming in the environment of intact motor neurons.

The mechanism by which an upper motor neuron lesion gives rise to microglial priming in the contralateral lumbar cord is not known. However, neurotransmission between upper and lower motor neurons of the corticospinal tract is glutamatergic [27,28] and it is known that ischemic neuronal injury results in the release of glutamate from neurons residing in the primary lesion site. This glutamatergic insult results in the subsequent activation of NMDA receptors on postsynaptic neurons with an increased calcium influx [29]. Increased extracellular glutamate is known to be associated with microglial activation and excitotoxic neuronal degeneration [30]. Treatment with the NMDA receptor antagonist MK-801 resulted in a decreased glial inflammatory response in the corticospinal tract of the spinal cord following MCAo in rats [22]. This strongly suggests that microglial activation is driven by signals from vulnerable lower motor neurons.

Several reports suggest that the physiological function of activated microglia is to either support and enhance the regeneration of neurons or, if neuronal damage is beyond repair, to elicit a response that results in the death of the neuron. These seemingly opposite outcomes beg the question of what controls the process of activation, and suggest that microglia may in fact be functionally different depending on whether they are "primed" or "activated". The function gained by the priming of microglia in the lumbar spinal cord following MCAo is not clear. Here we have shown that 24 hours and 72 hours post-reperfusion microglia have adopted a primed phenotype and not an amoeboid, phagocytic one. We speculate that in this state their role is protective and involves a heightened surveillance of glutamate levels and possibly the displacement of damaged upper motor neuron axon terminals from healthy lower motor neurons. A similar process has been described as "synaptic stripping" and involves the removal of presynaptic inputs from damaged post-synaptic neurons [31].

It would then be during this primed phase that the microglia may be serving as a protective entity to the vulnerable neuron. We have shown that during this time there is no significant loss of lower motor neurons, a fact that supports our hypothesis that microglia are not fully activated and phagocytic during this short period after occlusion. We have shown that these mice do have lesions caused by the occlusion that are predictable with behavioural testing (using the criteria developed by Belayev et al. [17]), and that the short time span of this occlusion does not contribute to significant mortality.

While the overall role of the microglia during the period directly following the occlusion was not the focus of this study, we present a model system that will allow for the examination of the roles of microglia when primed and not fully activated. Additionally, this model will allow for the process of transsynaptic injury, its relationship to microglial activation, and the signalling events that are involved during these processes to be studied in a reproducible, and predictable model system both in induced injury states and in murine disease models.

## Conclusion

We have determined that 30 minutes of MCAo was sufficient to induce a cortical lesion predictable with behavioural testing, with upper motor neuron involvement and a response of microglial priming in the contralateral lumbar spinal cord. Our findings strongly suggest that MCAo results in upper motor neuron degeneration and subsequent transsynaptic effects on the lower motor neuron environment resulting in perineuronal microglial priming, but not full microglial activation. This model will be useful in the study of transsynaptic injury-induced or disease-induced microglial priming and the role of such priming in the recovery from injury or during disease progression. The ability to predict lesion severity and the excellent survival rate make it suitable for testing differences in the neuronal and microglial response to transsynaptic injury in animals with pre-existing disease, including mouse models of neurodegenerative diseases with associated inflammatory processes involving microglial priming and activation such as ALS.

## Competing interests

The authors declare that they have no competing interests.

## Authors' contributions

KM carried out all animal care, neurobehavioural assessment, tissue collection, immunohistochemical studies, analysis, and drafting of the manuscript. IW and TH designed and performed the MCAo procedure. KV aided in the writing of the manuscript. MJS participated in the design of the experiment and the writing of the manuscript. All authors read and approved the final manuscript.
